# Pregnancy Induces an Immunological Memory Characterized by Maternal Immune Alterations Through Specific Genes Methylation

**DOI:** 10.3389/fimmu.2021.686676

**Published:** 2021-06-07

**Authors:** Xiaobo Huang, Liling Wang, Sijia Zhao, Hong Liu, Si Chen, Li Wu, Liping Liu, Jiahui Ding, Hengwen Yang, Anthony Maxwell, Zhinan Yin, Gil Mor, Aihua Liao

**Affiliations:** ^1^ Institute of Reproductive Health, Center for Reproductive Medicine, Tongji Medical College, Huazhong University of Science and Technology, Wuhan, China; ^2^ Hubei Province Engineering Research Center of Healthy Food, School of Biology and Pharmaceutical Engineering, Wuhan Polytechnic University, Wuhan, China; ^3^ Department of Obstetrics and Gynecology, Center for Reproductive Medicine, Anhui Province Hospital Affiliated to Anhui Medical University, Hefei, China; ^4^ Wuhan Women and Children Medical Care Center, Wuhan, China; ^5^ C.S. Mott Center for Human Growth and Development, Wayne State University School of Medicine, Detroit, MI, United States; ^6^ Zhuhai Institute of Translational Medicine, Zhuhai People’s Hospital Affiliated With Jinan University, Jinan University, Zhuhai, China; ^7^ The Biomedical Translational Research Institute, Faculty of Medical Science, Jinan University, Guangzhou, China

**Keywords:** pregnancy, immunological memory, epigenetic, preeclampsia, immune cells

## Abstract

During pregnancy, the maternal immune system undergoes major adaptive modifications that are necessary for the acceptance and protection of the fetus. It has been postulated that these modifications are temporary and limited to the time of pregnancy. Growing evidence suggests that pregnancy has a long-term impact on maternal health, especially among women with pregnancy complications, such as preeclampsia (PE). In addition, the presence of multiple immunological-associated changes in women that remain long after delivery has been reported. To explain these long-term modifications, we hypothesized that pregnancy induces long-term immunological memory with effects on maternal well-being. To test this hypothesis, we evaluated the immunological phenotype of circulating immune cells in women at least 1 year after a normal pregnancy and after pregnancy complicated by PE. Using multiparameter flow cytometry (FCM) and whole-genome bisulfite sequencing (WGBS), we demonstrate that pregnancy has a long-term effect on the maternal immune cell populations and that this effect differs between normal pregnancy and pregnancy complicated by PE; furthermore, these modifications are due to changes in the maternal methylation status of genes that are associated with T cell and NK cell differentiation and function. We propose the existence of an “immunological memory of pregnancy (IMOP)” as an evolutionary advantage for the success of future pregnancies and the proper adaptation to the microchimeric status established during pregnancy. Our findings demonstrate that the type of immune cell populations modified during pregnancy may have an impact on subsequent pregnancy and future maternal health.

## Introduction

Pregnancy is a period when the female body undergoes major transformations and physiological adaptations that are necessary to sustain and protect the growing fetus (1). These changes begin after conception and will affect several different organ systems in the mother, including the endocrine, cardiac, metabolic and immune systems (2). While some of these effects are transient, there are changes that will persist after parturition has occurred. Growing evidence suggests a relationship between childbearing patterns and maternal health outcomes (3). However, the underlying mechanism behind these effects and their long-term effects needs to be further elucidated.

The maternal immune system is one system that undergoes dramatic changes during the course of a pregnancy. The fetus is a combination of maternal and paternal antigens, who is considered as an allograft. Therefore, the mother’s immune system must undergo several modifications to preserve the developing fetus, while at the same time, serving as a strong protector against microbes as the fetus is especially sensitive to infections during the developmental period (4). For several years, it has been thought that the immunological modifications occur as transient changes and would return to normal after parturition. This concept originally stemmed from the idea that there is no biological need to maintain these changes, since the physiological interaction between the mother and fetus is terminated upon delivery of the placenta. However, there is strong evidence suggesting that maternal-fetal immune interaction is occurring before conception, during conception, and post conception (5). Before conception, the maternal immune system is exposed to paternal antigens in the semen, while post conception refers to the presence of fetal cells in the maternal tissues (6). Indeed, growing evidence suggests that maternal tissues get populated with fetal cells during pregnancy. The invasive fetal cells persist after pregnancy, which leaves the mother in a microchimeric state (5). Fetal Y-chromosome DNA can be detected in both cellular and cell-free compartments in maternal circulation beginning at 7 weeks of gestation (7). Additionally, the number of fetal cells in maternal tissue increases with the gestation, and these cells can still be found years after delivery (8). This microchimeric state requires the continued maintenance of adaptive changes in the maternal immune system in order to facilitate the long-term presence of the fetal cells.

In addition to the long-term adaptation to the fetal cells, the presence of an immunological memory has an evolutionary advantage for future pregnancies. Theoretically, the immunological memory that is maintained after each pregnancy will lead to better preparation for subsequent pregnancies. In a recent study by Gamliel et al. (9), they discovered a pregnancy-trained decidual natural killer (NK) cells with memory in repeated pregnancies. This unique NK cell subset expressed high level of NKG2C and LILRB1 and secreted high level of IFN-γ and VEGFα, which supported vascularization and can better support the subsequence pregnancies. The other observational evidence suggestive of the immune memory are in patients with pregnancy complications. Most pregnancy-related complications including preterm labor, placental abruption, preeclampsia (PE) and gestational diabetes appear to be resolved at delivery or shortly thereafter (10–13). Conversely, women who developed such pregnancy complications are known to be at an increased risk of developing similar complications in future pregnancies (3), which indicates the establishment of the pregnancy related memory.

A growing number of studies have shown that the pregnancy-specific complications continue to affect maternal health not only after parturition but also well beyond a woman’s reproductive years (14–16). A representative disease is PE. PE is an immune-mediated complication that can be life-threatening. It is characterized by hypertension after 20 weeks of gestation (17). The incidence of PE among pregnant women worldwide is 4%-5% (17). Moreover, the women experiencing PE are at a high incidence of cardiovascular disease and diabetes later in life and future pregnancy complications (18). Cheng et al. (19) raised the notion that chronic subclinical inflammation was related to the increased risk of adverse health outcomes in PE patients later in life, which was induced by microparticles, misfolded and aggregated proteins and nuclear and mitochondrial damage-associated molecular patterns. However, the exact underlying mechanism remains elucidated. Due to the long-term nature of many of these adverse outcomes, it is highly plausible that the alterations taking place during pregnancy may lead to specific epigenetic modifications responsible for the long-term effects.

Epigenetic modifications are involved in heritable regulation of genes without changing the underlying DNA sequence, which means the changes can be permanent in an individual. DNA methylation is indispensable for maintaining cell function and normal embryo growth and development (20). Epigenetic modifications are closely related to the evolution and function of the immune system. The modifications are deeply involved in the regulation of immune cell differentiation and function (21). However, whether pregnancy induced epigenetic modifications can regulate long-term adaptive alterations in the maternal immune cells is still unknown.

We tested the hypothesis that normal and abnormal pregnancies will induce epigenetic modifications that will result in changes in the maternal immune system, which can persist for years in the women. The objective of our study was to determine the presence of a pregnancy-induced immunological memory by characterizing the immunological changes in the peripheral blood of women at least 1 year after they had a previous normal pregnancy as well as women who had a previous pregnancy complicated by PE. Furthermore, we investigated whether epigenetic modifications are associated with long-term maternal immunological changes by characterizing the epigenetic changes in women with nulliparity, normal pregnancy and abnormal pregnancy complicated by PE at least 1 year postpartum.

**Figure 1:**
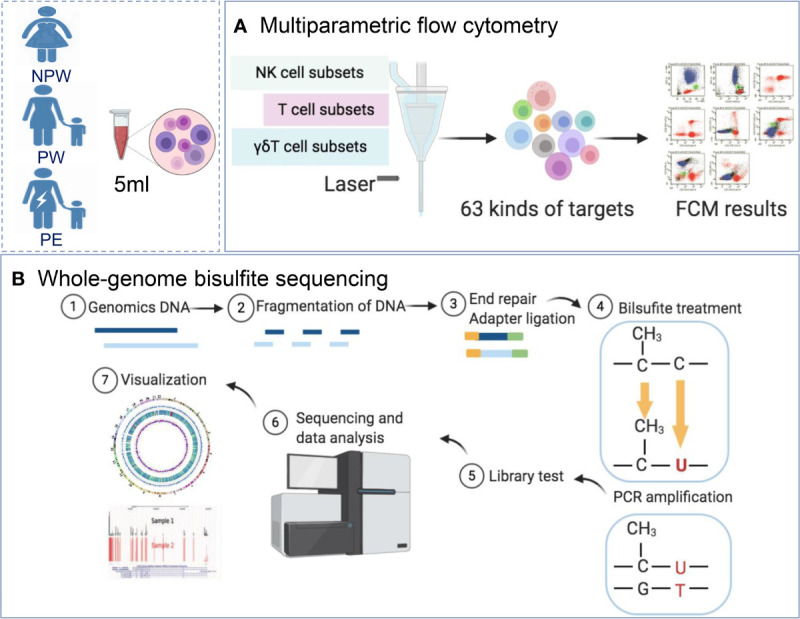
Study design. **(A)** Women were recruited into the NPW (n = 50), PW (n = 50) and PE (n = 14) groups. A total of 63 immunological parameters, including T cell, NK cell and γδT cell subsets in peripheral blood, were assessed using multiparameter flow cytometry. **(B)** Whole-blood DNA methylation in the NPW (n = 5), PW (n = 5) and PE (n = 5) groups was detected using WGBS. NPW, nonparous woman; PW, pregnant woman; PE, preeclampsia; WGBS, whole-genome bisulfite sequencing.

We used multiparameter flow cytometry (FCM) and whole-genome bisulfite sequencing (WGBS) (**Figure 1**) to demonstrate that pregnancy has long-term effects on the maternal immune system, which is characterized by immune cell subset modifications. These cellular subsets differed between normal pregnancy and pregnancy complicated by PE. Furthermore, immune cell subset modifications are due to the changes in the maternal methylation of specific genes that are associated with T cell and NK cell differentiation and function.

## Materials and Methods

### Patients and Samples

In total, 50 healthy nonparous women (NPW group), 50 women who had a normal pregnancy and were at least 1 year postpartum (PW group) and 14 women who had a pregnancy complicated by late-onset PE and were at least 1 year postpartum (PE group) were recruited between 2018 and 2019. The basic characteristics of all the subjects are summarized in **Table 1**. The history of PE during the last pregnancy was assessed according to ACOG guidelines (17). The late-onset PE is defined as PE that develops after 34 weeks of gestation. Women with fertility diseases, intrauterine contraceptive devices, autoimmune diseases, chronic infection, or fever were excluded for all the groups. Women in the PW and PE groups were recruited from the Center for Reproductive Medicine at Tongji Medical College of Huazhong University of Science and Technology (HUST) and the Department of Obstetrics and Gynecology at Maternal and Child Health Hospital of Wuhan, China. The women in the NPW group were volunteers from Tongji Medical College of HUST.

**Table 1:** Clinical characteristics of subjects.

Variable	NPW (n = 50)	PW (n = 50)	PE (n = 14)
Age (year)	24.12 ± 1.96	30.90 ± 3.01^***^	31.50 ± 3.70^***^
Menstrual period (day)	5.26 ± 0.85	5.36 ± 0.94	5.36 ± 1.01
Gestational frequency	-	1.44 ± 0.73	1.71 ± 0.99

Data are analyzed by Mann-Whitney U test, and presented as Mean ± SEM. Compared with NPW group: ***P<0.001. The age, menstrual period and gestational frequency of donors are shown in the table.

This study was approved by the Clinical Trial Ethics Committee of HUST (2018-S392). All experimental protocols were carried out in accordance with the approved guidelines and regulations. All the subjects gave written informed consents.

### Flow Cytometry

Whole-blood samples in 5 ml were collected in heparin-treated tubes from the three groups (NPW, PW and PE groups). Peripheral blood monocytes were isolated by Ficoll-Hypaque density gradient centrifugation (Pharmacia). After being washed twice with RPMI 1640 supplemented with 10% FCS (PAA laboratories), the cells were labeled with different fluorescein antibodies. All antibodies are listed in **Supplementary Table 1**. The experiments were performed according to the manufacturer’s instructions. Finally, a total of 63 immune cell subsets, including T cell subsets, NK cell subsets and γδT cell subsets, were determined by multiparametric FCM (**Supplementary Table 2**). The labeled cells were analyzed with BD FACSCanto™ flow cytometry (BD Biosciences, San Jose, CA, USA). Data were analyzed in FlowJo version 10 software (Tree Star, Ashland, OR, USA).

### Whole-Genome Bisulfite Sequencing

DNA was isolated from EDTA-treated blood (5 ml) with a TIANamp Blood DNA Midi Kit according to the manufacturer’s instructions. Genomic DNA degradation and contamination were monitored on agarose gels. The NanoPhotometer^®^ spectrophotometer (IMPLEN, CA, US) and Qubit^®^ 2.0 Fluorometer (Life Technologies, CA, USA) were used to assess the DNA purity and concentration.

### Library Construction and Sequencing

WGBS was performed as previously described (22). Briefly, a total of 5.2 μg genomic DNA spiked with 26 ng lambda DNA (Promega) was fragmented by sonication to 200-300 bp with Covaris S220, followed by end repair and adenylation. Then, these DNA fragments were treated twice with bisulfite using the EZ DNA Methylation-Gold™ Kit (Zymo Research) before the resulting single-strand DNA fragments were subjected to PCR amplification using KAPA HiFi HotStart Uracil + ReadyMix (2X) to recover enough DNA for sequencing. The prepared library was monitored by an Agilent 2100 Bioanalyzer (Agilent Technologies), quantified by quantitative PCR and then used for cluster generation. Libraries were sequenced using paired-end 150 bp reads on an Illumina HiSeq 2500/4000.

### Read Filtering and Alignment

Sequencing quality was assessed using FastQC (fastqc_v0.11.5, Babraham Bioinformatics, Cambridge, UK). Then, low-quality reads were filtered out with Trimmomatic software, and the remaining reads used in the subsequent analyses were counted as clean reads. Positional quality along the clean reads was confirmed to be QC > 30. A bisulfite-converted UCSC hg38 reference genome file was generated using Bowtie 2 (23), and the EpiGnome library sequence data were aligned to the reference genome. Sequence alignment of WGBS reads was performed using Bismark software (version 0.16.3). The sequencing depth and coverage were summarized using deduplicated reads.

### Estimating Methylation Level and Differential Methylation Analysis

Whether each cytosine was a methylated cytosine (mC) was determined using a binomial test. The sequence was divided into multiple bins that were 10 kb in size to calculate the methylation level. The sum of methylated and unmethylated read counts in each window was calculated. The methylation level for each window or C site shows the fraction of methylated Cs. Finally, the calculated methylation level was further corrected with the bisulfite nonconversion rate according to previous studies (24). DMRs were identified using DSS software (25).

### Gene Ontology Annotation and Pathway Enrichment Analysis of DMR-Related Genes

Gene Ontology (GO) analysis of genes containing DMRs was performed by the GOseq R package (version 3.6.3), in which gene length bias was corrected. GO terms with corrected P-values less than 0.05 were considered statistically significant. KOBAS software was used for Kyoto Encyclopedia of Genes and Genomes (KEGG) pathway enrichment analysis of DMR-related genes. Pathways with P-values less than 0.05 were determined to be statistically significant pathways.

### Statistical Analysis

The flow cytometry data are shown as the mean ± standard error of the mean (SEM) and were statistically analyzed using GraphPad Prism version 8.0 (GraphPad Software Inc., San Diego, CA, USA). Differences between the PW or PE groups and the NPW group were analyzed using the Mann–Whitney U test. A P value less than 0.05 was considered statistically significant.

## Results

### Effect of Pregnancy on Immune Cell Subsets

We first sought to determine the long-term effects that both normal and abnormal pregnancy had on specific subsets of maternal immune cells. We began to examine these subsets by collecting blood samples from three cohorts. The first group (referred to as PW) consisted of women who had delivered a healthy baby without any pregnancy complications and were +1 year postpartum. The second group (NPW) was made of women who were never pregnant. Lastly, the third group (PE) consisted of women who delivered a healthy baby while being affected with PE and were +1 year postpartum. Blood samples collected from each woman were analyzed with multiparameter flow cytometry (FCM) to assess 63 immunological parameters, including the T cell, NK cell and γδ T cell subsets (see **Supplementary Table 2** for the complete list). Specific gating strategies for the analysis of each subset are described in **Supplementary Figure 1**.

**Figure 2:**
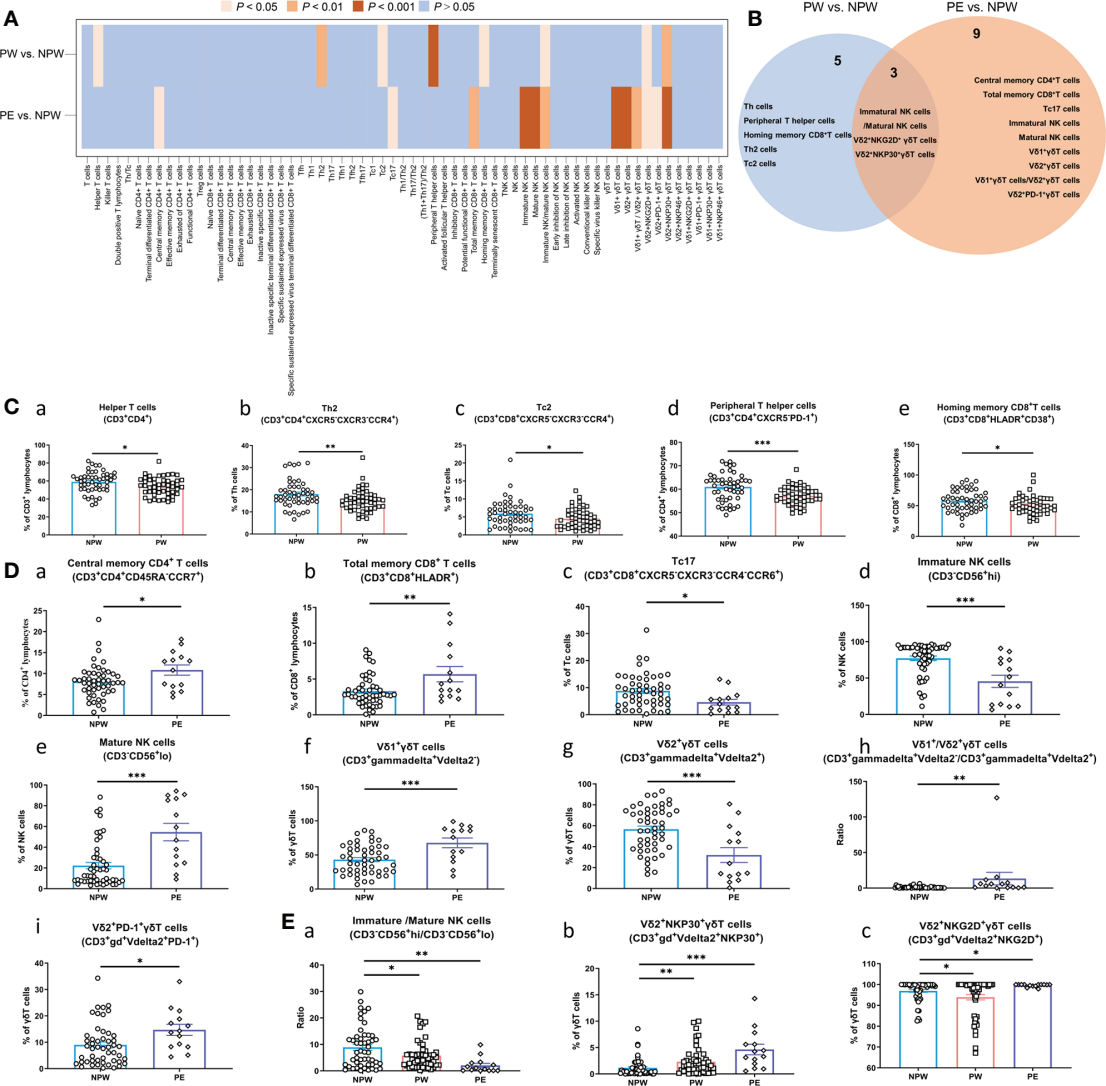
Immune modifications after normal or abnormal pregnancies. Lymphocyte composition and immunophenotypic characterization of T cells, NK cells and γδT cells in the NPW (n = 50), PW (n = 50) and PE (n = 14) groups were analyzed by multiparameter flow cytometry. **(A)** Heatmap showing the comparison of 63 immune parameters of T cells, NK cells and γδT cells in the PW and PE groups compared with the NPW group; **(B)** Venn diagram showing both unique and overlapping modified immune parameters in the PW and PE groups compared with the NPW group. **(C)** Changed immune cell subsets in women who experienced normal pregnancies **(a–e)**; **(D)** Changed immune cell subsets in women who experienced pregnancies complicated by PE **(a–i)**; **(E)** Common changed immune cell subsets in women who experienced pregnancy [normal (PW) or abnormal (PE)]. The Mann–Whitney *U* test was used to determine significant differences between the PW or PE groups and the NPW group **(a–c)**. All the data are shown as the mean ± SEM; *P < 0.05; **P < 0.01; ***P < 0.001.

Analysis of these samples showed major differences in immune cell composition between the PW and NPW groups. We showed different frequencies of 8 immune cell subsets between PW and NPW (**Figure 2A**). Additionally, we observed the changes in immune cell subset composition in the PE group. Although, the majority of these changes differed from those seen between the PW and NPW groups. Indeed, only 3 cell type modifications were common between the two pregnancy groups (**Figures 2A, B** and **Supplementary Table 3**).

Interestingly, we observed that normal pregnancy impacted the proportions of helper T cells (CD3^+^CD4^+^), T helper 2 (Th2) cells (CD3^+^CD4^+^CXCR5^-^CXCR3^-^CCR4^+^), cytotoxic T cell-2 (Tc2) cells (CD3^+^CD8^+^CXCR5^-^CXCR3^-^CCR4^+^), peripheral helper T cells (CD3^+^CD4^+^CXCR5^-^PD-1^+^), and homing memory CD8^+^ T cells (CD3^+^CD8^+^HLDAR^+^CD38^+^) (**Figures 2Ca–e**), which were significantly decreased in the PW group compared to the NPW group (*P* < 0.05). Next, we examined the ratios of immature/mature NK cells (CD3^-^CD56^+/hi^/CD3^-^CD56^+/lo^). This ratio was significantly decreased in PW compared to NPW (*P* < 0.05) (**Figure 2E a**). Finally, we observed an increase in the Vδ2^+^NKP30^+^ γδT cells (CD3^+^γδ^+^Vdelta2^+^NKP30^+^) (**Figure 2Eb**) and a decrease in the Vδ2^+^NKG2D^+^ γδT cells (CD3^+^γδ^+^Vdelta2^+^NKG2D^+^) (**Figure 2Ec**) in the PW group. These findings suggest that normal pregnancy changes the composition of immune cell populations and those changes are detectable +1 year after parturition.

Next, we wanted to examine how the immune cell profile was changed in women who had a previous (+1 year) pregnancy complicated with PE. We analyzed the immune cell composition of women in the PE group and found that 12 immune cell subsets were significantly different compared to NPW group. Interestingly, we also found that 3 of these subsets were similar to those observed in the PW group. Furthermore, we observed that the proportions of central memory T (CD4^+^ T_CM_) cells (CD3^+^CD4^+^CD45RA^-^CCR7^+^), total memory CD8^+^ T cells (CD3^+^ CD8^+^ HLA-DR^+^) were increased, and cytotoxic T cell-17 (Tc17) cells (CD3^+^CD8^+^CXCR5^-^CXCR3^-^CCR4^-^CCR6^+^) were decreased in the PE group compared to the NPW group (*P* < 0.001) (**Figures 2Da–c**). We further examined the γδ T cell populations in all 3 groups. We show an increased ratio of Vδ1^+^/Vδ2^+^ γδT cells (CD3^+^gammadelta^+^Vdelta2^-^/CD3^+^gammadelta^+^Vdelta2^+^) (*P* < 0.01); elevated proportion of Vδ1^+^ γδT cells (CD3^+^gammadelta^+^Vdelta2^-^) (*P* < 0.001), and a decreased proportion of Vδ2^+^ γδT cells (CD3^+^gammadelta^+^Vdelta2^+^) (*P* < 0.001) in the PE group (**Figures 2Df–h**). We additionally found a significantly decreased proportion of immature NK cells (CD3^-^CD56^+/hi^) (**Figure 2Dd**), an increased proportion of mature NK cells (CD3^-^CD56^+/lo^) (**Figure 2De**), and a decreased ratio of NK cells (CD3^-^CD56^+/hi^/CD3^-^CD56^+/lo^) (**Figure 2Ea**) in this same group. Similar to the PW group, the proportions of Vδ2^+^NKG2D^+^ γδT and Vδ2^+^NKP30^+^ γδT cells were affected in the PE group as well. However, women in the PE group showed an increased proportion of Vδ2^+^NKP30^+^ γδT cells (**Figure 2Eb**), while the proportion of Vδ2^+^NKG2D^+^ γδT cells was increased. This was essentially the opposite of what was seen in the PW group (**Figure 2Ec**). Altogether, this data suggests that pregnancy is inducing relatively long-term changes in the immune cell profiles of women. But the specific changes in these cell types are different between women who had normal pregnancies and women who had a pregnancy affected by PE.

### Epigenetic Modifications Induced by Pregnancy

Since we observed the changes in the immune cell composition that persisted several years (+1 years) upon completion of the pregnancy, we hypothesized that the observed changes may be due to epigenetic modifications taking place during the pregnancy. To test this hypothesis, we performed whole-genome DNA methylation profile analysis with peripheral blood obtained from women who had their first pregnancy within the last 1 to 2 years. Total 15 women were recruited into this study according to the same sample collecting rule in the FCM part and were divided into three groups. The first group consisted of samples collected from 5 women (PW) who had only one normal pregnancy at least 1 years postpartum. The second group consisted of 5 women who had only one pregnancy complicated by preeclampsia (PE) at least 1 years postpartum. The final group consisted of women who never had a pregnancy before (NPW).

**Figure 3:**
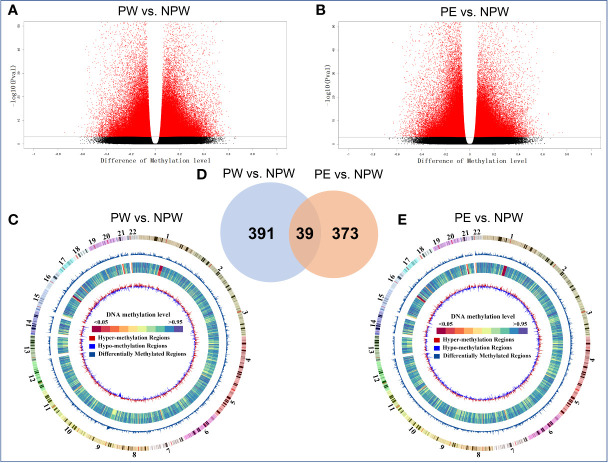
Whole-genome DNA methylation profiles of the PW and PE groups. **(A, B)** Methylated sites of PW and PE groups in a volcano plot. Most detected sites were methylated in immune cells in the PW and PE groups compared with the NPW group. Red and black plots represent significantly differentially and not differentially methylated sites, respectively. The dots on the left and right sides of each volcano represent hypo- and hypermethylation levels. **(C, E)** Circus plot of methylation levels in the PW and PE groups compared with the NPW group. There are five circles from the outer side to the inner side, which represent the methylation in the chromosome, DMR, mean methylation levels in the NPW group and PW group, and hypo- and hypermethylation distribution in the PW group compared with the NPW group. Red represents the hypomethylated region, and blue represents the hypermethylated region. **(A, C)** PW *vs*. NPW. **(B, E)** PE *vs*. NPW. *P* < 0.05. **(D)** The 430 DMGs that occurred after normal pregnancy are shown in blue (PW *vs*. NPW), and the 412 DMGs after PE are shown in yellow (PE *vs*. NPW). A total of 39 common genes were methylated after normal pregnancy and PE and are shown in the blue-yellow overlap. **(A)** PW *vs*. NPW. **(B)** PE *vs*. NPW. *P* < 0.05.

As shown in the volcano plot in **Figures 3A, B**, we observed that women in the PW group as well as women in the PE group had a major impact on the methylation status of multiple genes that are associated with peripheral immune cells. Hypo- and hypermethylation was observed in both pregnancy groups compared to NPW. This methylation primarily occurred on different chromosomes (chr), mainly chr 1, chr 11, chr 16, chr 17, chr21 and chr22 (**Figures 3C–E**).

Next, we compared the number of differentially methylated genes (DMGs) within the observed differentially methylated regions (DMR) and observed major differences between PW and PE. A total of 430 DMGs were identified in the PW group and 412 in the PE group. Using Venn analysis, we observed 39 common DMGs between the two pregnancy groups (**Figure 3D**). This data suggested that pregnancy has an impact on the methylation status of genes associated with the maternal immune system. While there is some overlap in DMGs in the two pregnancy groups, major differences still exist. This finding further suggests that normal and abnormal pregnancy leads to the changes in immune cell populations, which may be facilitated by epigenetic modifications that are induced through different processes associated with pregnancy.

### Gene Ontology Analysis of DMGs in the PW and PE Groups

**Figure 4:**
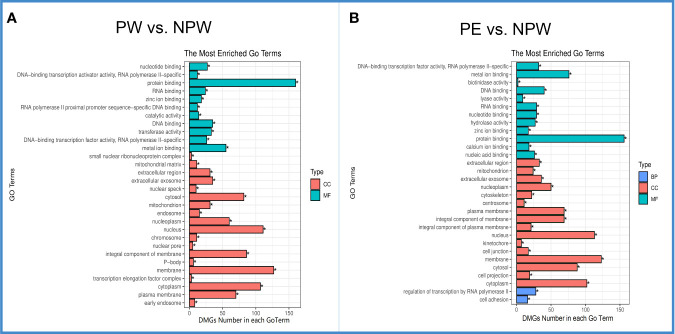
Gene ontology (GO) of DMGs in the PW and PE groups. **(A)** The top 30 significantly different GO terms in the PW group were mainly in the cell component (CC) and molecular function (MF) categories. Green represents MF, and red represents CC. **(B)** The top 30 significantly differentially expressed GO terms in the PE group were in the CC, MF and biological process (BP) categories. Red represents CC, blue BP and green MF. *q < 0.05.

To better understand how the DMGs seen in this data are involved in modulating immune cell populations after pregnancy, we examined what biological processes they were involved in *via* GO analysis. DMGs were enriched in a total of 2097 related GO terms in the PW group. As shown in **Figure 4A**, the top 30 significantly different GO terms (*q* < 0.05) were in the cell component (CC) (19) and molecular function (MF) categories (11). The most enriched CC terms (DMG number ≥ 50) were cytosol, nucleoplasm, nucleus, integral component of membrane, membrane, cytoplasm and plasma membrane (**Figure 4A** in red), and the most enriched MFs (DMG number ≥ 50) were protein binding and metal ion binding (**Figure 4A** in green).

In the PE group, DMGs were enriched in a total of 2138 related GO terms. Sixteen were CC terms and 12 were MF terms. Interestingly, the most enriched GO terms in the CC and MF categories in the PE complicated pregnancies were similar to those observed in the PW group (**Figures 4A, B**). However, in the PE group, the top 30 significantly different GO terms (*q* < 0.05) included 2 different biological process (BP): regulation of transcription by RNA polymerase II and cell adhesion (**Figure 4B** in blue).

### KEGG Enrichment of DMGs in the PW and PE Groups

**Figure 5:**
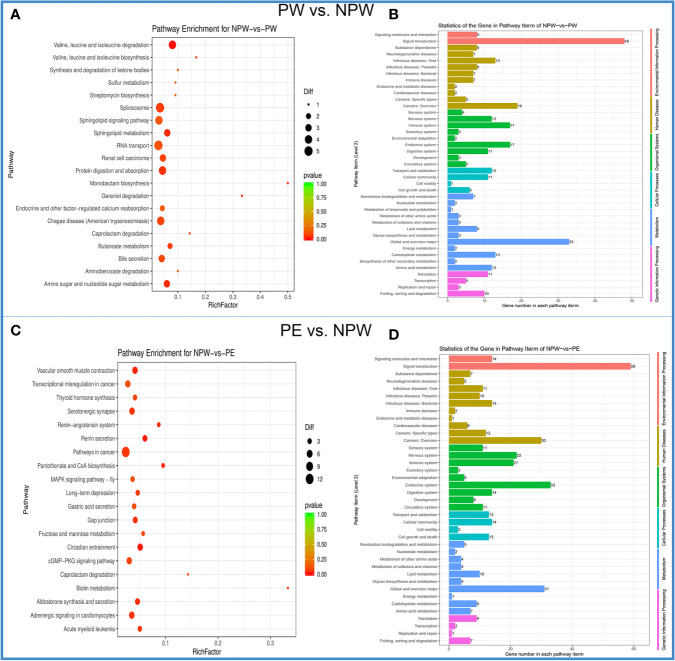
Kyoto Encyclopedia of Genes and Genomes (KEGG) enrichment of DMGs in the PW and PE groups. **(A)** The scatter plot of the KEGG signaling pathway for PW *vs*. NPW. The ordinate represents the degree of enrichment, while the abscissa represents 20 KEGG signaling pathways. The black dots indicate the number of genes contained in the signal pathway, and the color of the dots represents the size of the *P* value. The reddish color indicates a stronger correlation. **(B)** KEGG signaling pathway histogram. The ordinate on the left represents 41 KEGG signal pathways, and the abscissa demonstrates the statistics of genes in each pathway term for PW *vs*. NPW. The ordinate in the left and color represents the different first-level KEGG pathways. **(C)** The scatter plot of the KEGG signaling pathway for PE *vs*. NPW. The ordinate represents the degree of enrichment, while the abscissa represents 20 KEGG signaling pathways. The black dots indicate the number of genes contained in the signaling pathway, and the color of the dots represents the size of the *P* value. The reddish color indicates a stronger correlation. **(D)** KEGG signaling pathway histogram. The ordinate on the left represents 41 KEGG signaling pathways, and the abscissa demonstrates the statistics of genes in each pathway term for PE *vs*. NPW. The ordinate in the left and color represent the different first-level KEGG pathways.

From a total of 201 KEGG signaling pathways analyzed, we found 6 specific signaling pathways that were significantly modified in the PW group (**Figure 5A**). They are mainly associated to metabolic adaptation: valine, leucine, and isoleucine degradation, monobactam biosynthesis, sphingolipid metabolism, amino sugar and nucleotide sugar metabolism, geraniol degradation, and protein digestion and absorption (**Figures 5A, B**).

In the PE group, we identified 10 specific signaling pathways that were significantly modified. The differential KEGG enrichment pathways in the PE group mainly consisted of circadian entrainment, renin secretion, vascular smooth muscle contraction, aldosterone synthesis and secretion, pantothenate and CoA biosynthesis, biotin metabolism, renin-angiotensin system, gap junction, serotonergic synapse and acute myeloid leukemia (**Figures 5C, D**). These findings suggest that the DMGs in pregnancy are distributed in different KEGG pathways and are different between PW and PE.

### Methylation Levels of Significant DMGs in Each KEGG Pathway

**Figure 6:**
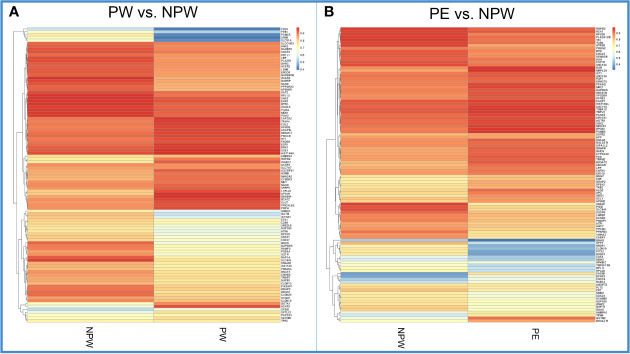
Heatmap of methylation levels of significant DMGs in each KEGG pathway in the PW and PE groups. **(A)** Methylation levels of the significant DMGs in 201 KEGG pathways in the PW and NPW groups. Blue color represents lower methylation levels. Red represents higher methylation levels. **(B)** Methylation levels of the significant DMGs in 196 KEGG pathways in the PE and NPW groups. Blue color represents lower methylation levels. Red represents higher methylation levels.

We also analyzed the methylation levels of specific genes associated with each KEGG pathway in women in the PW and PE groups. We found significantly different hypo- or hypermethylated genes in 201 KEGG pathways, which are summarized in the heat map shown in **Figures 6A, B**.

**Figure 7:**
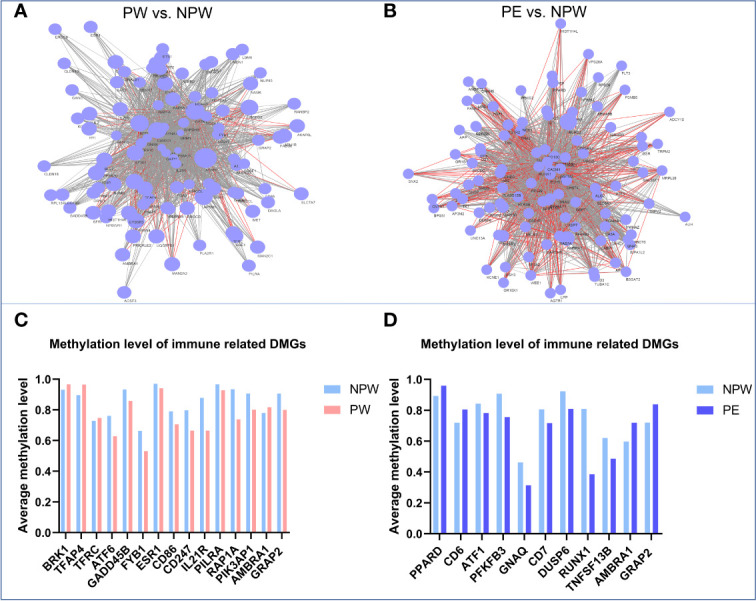
Network diagram of methylation levels of significant DMGs and cell subset modification related DMGs in each KEGG pathway in the PW and PE groups. **(A)** Methylation levels of the significant DMGs in 201 KEGG pathways in the PW group compared to those in the NPW group. The larger the node is, the higher the methylation level of the genes in the PW group compared to the NPW group. The gray line between two genes represents the near-identical methylation changes (both increased and decreased) between the two compared groups, while the red line represents opposite methylation changes between the groups. **(B)** Methylation levels of the significant DMGs in 196 KEGG pathways in the PE group compared to the NPW group. The larger the node is, the higher the methylation level of the gene in the PE group compared to the NPW group. The gray line between two genes represents the near-identical methylation changes (both increased and decreased) between the two compared groups, while the red line represents opposite methylation changes (one hypermethylated gene and one hypomethylated) between the two compared groups. **(C)** Average methylation levels of T cell- and NK cell-related DMGs in the PW and NPW groups. **(D)** Average methylation level of T cell-related DMGs in the PE and NPW groups.

In the network diagram shown in **Figure 7**, we can observe that pregnancy promotes methylation changes characterized by either hypo- or hypermethylated regions (**Figures 7A, B**). Some of these changes are common to both pregnancy groups. However, there are specific methylation areas that differ when the pregnancy is healthy or complicated by PE (**Figure 7**). In the **Supplementary Data**, we provide a full list of the areas that are differentially methylated between women who had normal or those who had pregnancies complicated by PE (**Supplementary Table 4**).

### The Relationship Between DMGs and Cell Subset Modification Induced by Pregnancy

To determine whether the DMGs that were found in KEGG pathways have connections with immune cell subset modifications that were induced by a normal or abnormal pregnancy, we searched for the functions of 430 DMGs identified in the PW group and 412 DMGs in the PE group *via* the NCBI gene library. As expected, most DMGs are related to immunological functions (**Figure 7**). We found 3 hypermethylated genes (*BRK1*, *TFAP4* and *TFRC*) and 10 hypomethylated genes (*ATF6*, *GADD45B*, *FYB1*, *ESR1*, *CD86*, *CD247*, *IL21R*, *PILRA*, *RAP1A* and *PI3KAP1*) in the PW group. These genes are associated with the differentiation and function of T cells and NK cells (26–38) (**Figure 7C**). Among the DMGs in the PE group, we identified 2 hypermethylated genes (*PPARD* and *CD6*) and 7 hypomethylated genes (*ATF1*, *PFKFB3*, *GNAQ*, *CD7*, *DUSP6*, *RUNX1* and *TNFSF13B*). These genes have regulatory roles in T cell subset differentiation and function (39–47) (**Figure 7D**). Interestingly, the two DMGs found in both the PW and PE groups, *AMBRA1* and *GRAP2*, are closely associated with TCR signal-induced T cell modification and homeostasis. Compared to that in the control group, *AMBRA1* was hypermethylated in both the PW and PE groups. However, *GRAP2* was hypomethylated in the PW group but hypermethylated in the PE group (**Figures 7C, D**).

In summary, the identified gene modifications, characterized by hyper- or hypomethylated genes, may be responsible for the long-term effects that normal and abnormal pregnancies have on immune cell subset composition.

## Discussion

In this study, we described for the first time the long-term effect of pregnancy on the maternal immune system and provide the evidence for the existence of a long-term “immunological memory of pregnancy (IMOP)”. We used multiparameter FCM and WGBS to detect the long-term alterations in immune cell subsets and DNA methylation of peripheral blood from women who had a prior (at least 1 years) normal pregnancy or pregnancy complicated by PE. We found that specific immune cell populations of T cells, NK cells and γδ T cells were modified one year or more after parturition. Furthermore, there was marked epigenetic modification that persisted for more than 1 years in the circulating immune cells of women who had previous pregnancies. Epigenetic modifications occurred regardless of whether the pregnancy was normal or abnormal.

During pregnancy, the maternal immune system undergoes significant modifications in order to adapt to and protect the fetus, which requires the establishment and maintenance of immune tolerance while efficiently responding to infections at the same time (1). The process of adaptation involves the modulation of several immune cell subpopulations, particularly T cells, B cells and NK cells, which coordinate the establishment of a delicate immunological balance at the maternal and fetal interface (48).

Surprisingly, the alterations of certain immune cell populations due to pregnancy can persist for a long time after delivery. In a recent study by Kierffer et al. (49), they found that the proportions of peripheral CD4^+^ CM (CD45RO^+^CCR7^+^), CD4^+^ effector memory (EM, CD45RO^+^CCR7^-^) and activated (CD69^+^) memory T cells in parous women at average 18 months postpartum were significantly higher than those in nulligravid women, suggesting a persistent influence of pregnancy on the memory T cell populations. However, the activation status of CD8^+^ memory cells did not differ between the two groups. The same team further found that activated proportions of total CD4^+^ memory, CD4^+^ EM, and CD4^+^ CM were lower in formerly preeclamptic women than those in formerly healthy pregnant women (18). The authors suggested that memory CD4^+^ T cell populations might involve in PE and its recurrence risk more likely. In the current study, we found that the proportions of central memory T (CD4^+^ T_CM_) cells (CD3^+^CD4^+^CD45RA^-^CCR7^+^) and total memory CD8^+^ T cells (CD3^+^CD8^+^HLA-DR^+^) after at least 1 year postpartum were significantly higher in the PE group than those in the NPW group. Although our data are not completely consistent with the previous study, the evidence supports that formerly healthy or abnormal pregnancy may leave the maternal immune system with different imprint. However, whether the pregnancy-induced alternations in memory CD4^+^ or CD8^+^ T cells affect the subsequent pregnant outcomes still need further investigations in a large longitude study.

Interestingly, we found common immunological changes that were maintained between women who had normal pregnancies and those who had pregnancies complicated by PE, such as the changes in the ratio of immature NK/mature NK cells, and the proportions of Vδ2^+^NKG2D^+^ γδ T cells and Vδ2^+^NKP30^+^ γδ T cells. However, there were some specific immunological changes associated with abnormal pregnancies. We observed that after normal pregnancy, the proportion of Vδ2^+^NKG2D^+^ γδ T cells decreased, but increased after PE. NKG2D, an activating receptor for NK cells, can directly trigger TNF-α production and release cytolytic granules in Vδ2^+^ T cells, without antigen-dependent fashion (50). A recent study identified several unique T cell subsets in intraepithelial lymphocytes of Crohn’s disease, including RORγt positive NKp30^+^ γδ T cells. Upon NKp30 engagement, these cells produced IL-26 with antibacterial properties, indicating the possible protective role in intestinal homeostasis (51). To the best of our knowledge, there are no reports on the γδ T cell alternations postpartum, which made us unable to compare our data to them.

Basing on our findings, we postulated that pregnancy-induced immunological memory, namely IMOP, might be necessary for two aspects: 1) preparation for consecutive pregnancies, as a healthy pregnancy not only impacts the ongoing pregnancy but may impact the oncoming pregnancies, and 2) immunological priming for the resulting microchimerism status of pregnancy. Considering that women can become pregnant multiple times, the IMOP in relation to the next pregnancy may confer an evolutionary advantage, because pregnancy primes fetal-specific immunity capable of alloimmunization and tolerogenic phenotypes that are maintained for the next pregnancy (52). In addition, IMOP is required for the acceptance of fetal cells transferred to the maternal circulation that persists in the mother for decades after giving birth. Fetal cells are present in the maternal peripheral blood as early as 7 weeks of gestation during which fetal Y-chromosomal DNA can be detected. This effect can persist 27 years after giving birth to a son (7, 53). Notably, the retention of fetal cells in maternal tissues is not limited to humans, but it is highly conserved across mammalian species, which might suggest a biological benefit in establishing the IMOP that drives the maintenance of tolerance to fetal antigens in mothers after pregnancy (8).

On the other hand, PE establishes a different IMOP that is characterized by increased inflammatory immune cells, which are a main immune factor contributing to the onset of PE. PE-derived immunological memory might be responsible for the increased risks of PE in subsequent pregnancies (12). In addition, women with PE are more likely to develop hypertension later in life and even cardiovascular problems.

A longitude study which analyzed the cellular transcriptome dynamics of the whole blood sample from 49 pregnant women, found that multiple biological processes and pathways are related to immunity, and specific genes changing during pregnancy are closely related to CD4^+^, CD8^+^ T cells and CD56^+^ NK cells (54). We then asked how the IMOP is established. Methylation analysis of immune cells isolated from peripheral blood of women who had a previous pregnancy revealed major epigenetic modifications. These modifications are associated with regulatory effectors on gene activity, while not changing the underlying DNA sequence. The results of the current study demonstrate that the epigenetic modifications are associated with genes that are enriched in different GO and KEGG pathways. Among DMGs in women who had normal pregnancies, we found that DMGs were mainly enriched in the sphingolipid metabolism pathway. A previous study demonstrated that sphingosine-1-phosphate and its synthetase-sphingosine kinase in sphingolipid metabolism pathway are closely related to the differentiation and invasion of trophoblasts and placental angiogenesis, which are important for pregnancy (55). Among DMGs in women who had pregnancies complicated by PE, we found that DMGs were mainly enriched in the renin-angiotensin system. Angiotensin II works through angiotensin receptors 1 (AT1) and AT2 receptors. AT1 is found in syncytiotrophoblasts, and AT2 plays important role in fetal development, which can inhibit cell growth, increase apoptosis, cause vasodilation and regulate fetal tissue development (56).

Furthermore, in this study, we demonstrated that these epigenetic modifications are associated with genes that control the differentiation of immune cells. Among DMGs in women who had normal pregnancies, we found genes closely related to the development of immune cells, such as *BRK1* and adhesion and degranulation-promoting adapter protein (ADAP). *BRK1* depletion in CD3^+^ T cells contributes to the inhibition of T cell activation and actin polymerization accompanied by defects in immunological synapse formation (26). ADAP (encoded by FYB1 and formerly known as SLAP-130 or FYB) is a hematopoietic-specific adapter that is required for efficient TCR signaling, T cell activation, differentiation, proliferation and adhesion (57). ADAP regulates T cell activation by promoting Ag-dependent T cell-APC interactions, resulting in enhanced T cell sensitivity to Ag, and by participating in T cell survival (58).

In addition to T cells, ADAP also has a regulatory role in inflammatory cytokine production, NK cell cytotoxicity and migration (59, 60). A recent study showed that ADAP-deficient NK cells have impaired cytotoxic capacity and migration defects, as indicated by reduced CD107a surface expression, inefficient perforin production, and reduced expression of integrin CD11a compared to wild-type NK cells during pathogen challenge (60). Among DMGs in women who had pregnancies complicated by PE, we also found genes closely related to the development of immune cells, such as peroxisome proliferator-activated receptor delta (PPARD) and 6-Phosphofructo-2-Kinase/Fructose-2,6-Bisphosphatase 3 (PFKFB3). PPARD is a member of the PPARs family which plays critical roles in regulating glucose and lipid metabolism. They are mediators of inflammation and angiogenesis which are involved in the maternal adaptation dynamics during pregnancy to serve the requirements of the growing fetus (39). Besides, PPARs are also immune regulators both in the innate and adaptive immune system (61). PFKFB3 is regulated by the PI3K/Act signaling pathway in activated T lymphocytes. PFKFB3 deficiency can cause the energy-deprived and autophagy-deficient, apoptosis-sensitive T-lymphocytes which are characteristic of autoimmune disease (42).

**Figure 8:**
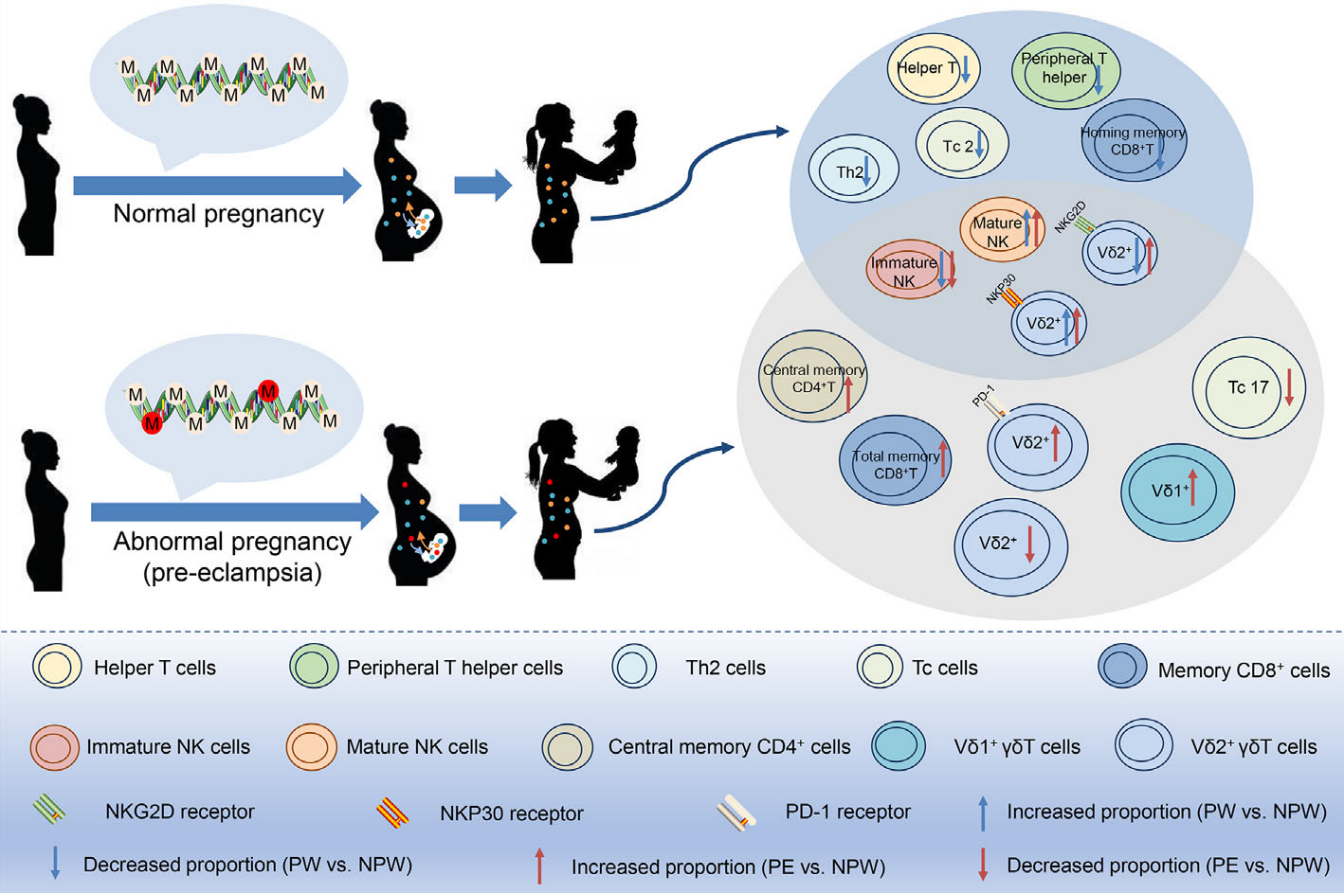
Pregnancy-mediated DNA methylation modification may contribute to the long-term impact of normal and abnormal pregnancies on maternal peripheral immune cell subsets. In normal or preeclamptic pregnancies that resulted in the birth of a healthy baby, methylation modifications in peripheral immune cells were different and persisted at least one year postpartum, as well as did differences in the immune cell subsets including T, NK and γδT cell subsets. Therefore, we speculate that pregnancy-induced differential methylation of genes in peripheral blood may be related to the altered proportion of immune cell subsets. Red circles in DNA: epigenetic changes in abnormal pregnancy.

In the current study, we propose that both normal and abnormal pregnancies induce immunological memory based on specific epigenetic modifications that are maintained in preparation for the next pregnancy, which might provide an evolutionary advantage. Furthermore, this immunological memory might allow the protection of chimeric fetal cells that have been incorporated into the maternal circulation. Consequently, in contrast to a pregnancy with complications, a healthy pregnancy will have long-term advantages to maternal health (**Figure 8**). Understanding the characteristics of pregnancy-induced immunological memory is essential for the prevention and management of subsequent pregnancies as well as future maternal health.

## Study Strength and Limitations

The strengths of our study included the first time report the existence of an immunological memory established during pregnancy that is detected at least 1 years postpartum. The design of our study allowed us not solely focuses on the immunological memory of women who have experienced a successful normal pregnancy. Our study also focuses on the immunological memory of women who have experienced an abnormal pregnancy (PE). The current data supporting the concept of IMOP may explain clinical observations in patients with consecutive pregnancy complications. Our study not only focused on the cellular changes, but also described the molecular mechanisms responsible for the cellular changes. We postulate and demonstrate that pregnancy has a long-term programing effect on the maternal immune system by promoting epigenetic modifications. Importantly, we connected the changes of immune cells with epigenetics to explain the molecular mechanism establishing the immunological memory.

The limitations of this study are the small sample size in PE group and not a longitudinal study. In the current study, the comparison was done between the women without any pregnancy and those formerly experiencing normal or preeclamptic pregnancies. Although there were differences in the ages between NPW and PW/PE groups: (24.12 ± 1.96) y in NPW group, (30.90 ± 3.01) y in PW (*P* < 0.001) and (31.50 ± 3.70) y in PE (*P* < 0.001), it is not a factor associated with the immunological differences. Vasson et al. (62) studied the immune biomarkers including peripheral blood immune cell subsets among 300 healthy people at 20-75 years old, aiming to explore the effects of ages on the immune system. They found that the proportions of CD4^+^ T cells and CD8^+^ T cells had no difference between 20-29 y and 30-39 y. Consequently, the difference of ages does not represent a bias on the proportions of immune cell subsets in the present study. However, our findings strongly encourage the need to perform a longitude study which would determine the specific immunological modification before, during and after normal pregnancies as well as pregnancy complications. Moreover, other influential factors including environmental factors, intermittent exposure to infections, socio-economic factors, stress and changing the partner during subsequent pregnancies will be also considered.

## Data Availability Statement

The WGBS data have been deposited into the National Center for Biotechnology Information Sequence Read Achieve database (accession no. PRJNA730590).

## Ethics Statement

The studies involving human participants were reviewed and approved by The Clinical Trial Ethics Committee of HUST. The patients/participants provided their written informed consent to participate in this study.

## Author Contributions

XH, ZY, GM, and AL. conceived and designed the experiments. XH, LWu, LL, and AL conducted donor recruitment and collected all relevant clinical information. XH, SC, and HY performed data analysis. XH, SZ, and AL design the figures. XH, LWa, SZ, HL, and JD wrote the manuscript. GM, AM, and AL edited the manuscript. ZY, GM, and AL supervised this work. All authors contributed to the article and approved the submitted version.

## Funding

The study was supported by National Key Research & Developmental Program of China (2018YFC1003900; 2018YFC1003904); NIAID (1R01AI145829-01).

## Conflict of Interest

The authors declare that the research was conducted in the absence of any commercial or financial relationships that could be construed as a potential conflict of interest.

## Supplementary Material

Supplementary Figure 1 Gating strategy of flow cytometric analysis. **(A)** Gating of the CD4^+^ T, CD8^+^ T, NK, NKT and γδ T cell subsets. **(B)** Gating of the CD4^+^ Treg, CD4^+^ T and CD8^+^ T subsets based on the expression of CD45RA and CCR7. **(C, D)** Gating of the Th, Tfh and Tc cell subsets. **(E)** Gating of the NK cell subsets. **(F)** Gating of the γδ T and Vδ2^+^ γδT cell subsets.
