# Supplementary figures and images for: Pregnancy Induces an Immunological Memory Characterized by Maternal Immune Alterations Through Specific Genes Methylation
Source: Front Immunol. 2021 Jun 7;12:686676. doi: 10.3389/fimmu.2021.686676 (PMC8215664; doi:10.3389/fimmu.2021.686676)

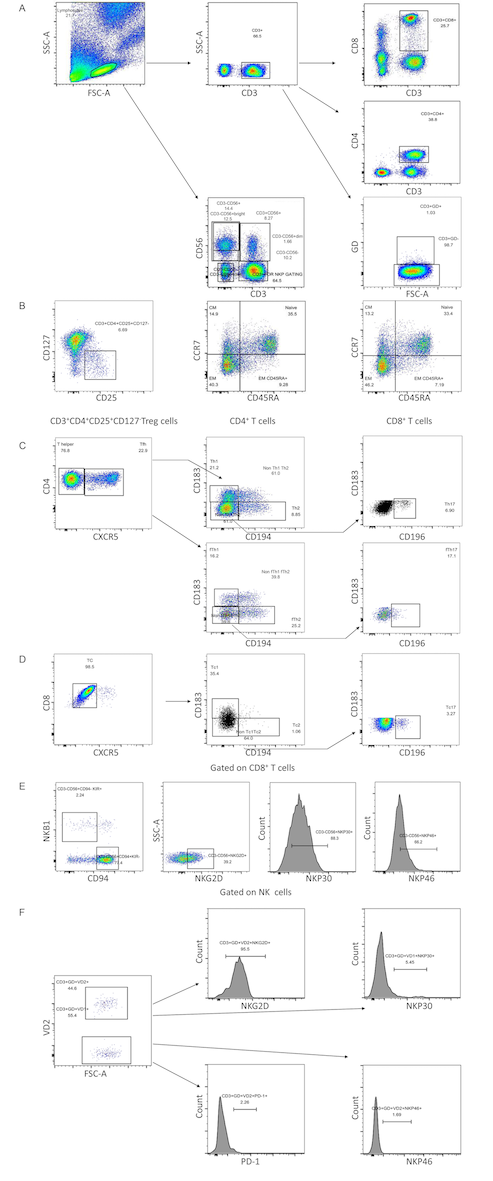

Supplement: Supplementary Figure 1 — Gating strategy of flow cytometric analysis. (A) Gating of the CD4+ T, CD8+ T, NK, NKT and γδ T cell subsets. (B) Gating of the CD4+ Treg, CD4+ T and CD8+ T subsets based on the expression of CD45RA and CCR7. (C, D) Gating of the Th, Tfh and Tc cell subsets. (E) Gating of the NK cell subsets. (F) Gating of the γδ T and Vδ2+ γδT cell subsets. [file Image_1.tiff]
