# Supplementary material for: Pregnancy Induces an Immunological Memory Characterized by Maternal Immune Alterations Through Specific Genes Methylation
Source: Front Immunol. 2021 Jun 7;12:686676. doi: 10.3389/fimmu.2021.686676 (PMC8215664; doi:10.3389/fimmu.2021.686676)
Supplement: Supplementary file 2 [file Table_1.docx]

**Table S1. Antibodies used in flow cytometry staining**

| **Antibody** | **Identifier** | **Source** |
| --- | --- | --- |
| PerCP-Cy5.5-conjugated anti-human CD3 antibody | Clone ID: UCHT1, Cat# 560835 | BD Biosciences |
| PE-Cy7-conjugated anti-human CD28 antibody | Clone ID: CD28.2, Cat# 560684 | BD Biosciences |
| BV510-conjugated anti-human CD8 antibody | Clone ID: SK1, Cat# 563919 | BD Biosciences |
| APC-H7-conjugated anti-human CD4 antibody | Clone ID: RPA-T4, Cat# 560158 | BD Biosciences |
| BV421-conjugated anti-human CD127 antibody | Clone ID: HIL-7R-M21, Cat# 562436 | BD Biosciences |
| AF647-conjugated anti-human CCR7(CD197) antibody | Clone ID: 3D12, Cat# 557734 | BD Biosciences |
| PE-conjugated anti-human CD25 antibody | Clone ID: M-A251, Cat# 555432 | BD Biosciences |
| FITC-conjugated anti-human CD45RA antibody | Clone ID: HI100, Cat# 555488 | BD Biosciences |
| FITC-conjugated anti-human CD8 antibody | Clone ID: HIT8α, Cat# 555634 | BD Biosciences |
| BV421-conjugated anti-human CD57 antibody | Clone ID: NK-1, Cat# 563896 | BD Biosciences |
| BV510-conjugated anti-human CD38 antibody | Clone ID: HIT2, Cat# 563251 | BD Biosciences |
| AF647- conjugated anti-human CD279(PD-1) antibody | Clone ID: EH12.1, Cat# 560838 | BD Biosciences |
| PE-conjugated anti-human HLA-DR antibody | Clone ID: G46-6, Cat# 555812 | BD Biosciences |
| APC-H7-conjugated anti-human CD3 antibody | Clone ID: SK7, Cat# 560176 | BD Biosciences |
| PE-Cy7-conjugated anti-human CD314(NKG2D) antibody | Clone ID: 1D11, Cat# 562365 | BD Biosciences |
| AF647-conjugated anti-human CD337(NKP30) antibody | Clone ID: p30-15, Cat# 558408 | BD Biosciences |
| BV510-conjugated anti-human CD335(NKP46) antibody | Clone ID: 9E2 / NKp46, Cat# 564064 | BD Biosciences |
| BV421-conjugated anti-human CD56 antibody | Clone ID: NCAM16.2, Cat# 562751 | BD Biosciences |
| PerCP-Cy5.5-conjugated anti-human CD94 antibody | Clone ID: HP-3D9, Cat# 562361 | BD Biosciences |
| PE-conjugated anti-human NKB1 antibody | Clone ID: DX9, Cat# 555967 | BD Biosciences |
| BV421-conjugated anti-human TCRγδ antibody | Clone ID: B1, Cat# 562560 | BD Biosciences |
| BB515-conjugated anti-human CD279(PD-1) antibody | Clone ID: EH12.1, Cat# 564494 | BD Biosciences |
| PE-conjugated anti-human Vδ2 TCR antibody | Clone ID: B6, Cat# 555739 | BD Biosciences |
| PE-Cy7-conjugated anti-human CD4 antibody | Clone ID: SK3, Cat# 557852 | BD Biosciences |
| PerCP-Cy5.5-conjugated anti-human CD8 antibody | Clone ID: SK1, Cat# 565310 | BD Biosciences |
| AF488-conjugated anti-human CD183 antibody | Clone ID: 1C6 / CXCR3, Cat# 558047 | BD Biosciences |
| BV510-conjugated anti-human CD196(CCR6) antibody | Clone ID: 11A9, Cat# 563241 | BD Biosciences |
| AF 647-conjugated anti-human CXCR5(CD185) antibody | Clone ID: RF8B2, Cat# 558113 | BD Biosciences |
| BV421-conjugated anti-human CD194 antibody | Clone ID: 1G1, Cat# 562579 | BD Biosciences |
| PE-conjugated anti-human CD279 antibody | Clone ID: MIH4, Cat# 557946 | BD Biosciences |
