# Supplementary material for: Pregnancy Induces an Immunological Memory Characterized by Maternal Immune Alterations Through Specific Genes Methylation
Source: Front Immunol. 2021 Jun 7;12:686676. doi: 10.3389/fimmu.2021.686676 (PMC8215664; doi:10.3389/fimmu.2021.686676)
Supplement: Supplementary file 3 [file Table_2.docx]

**Table S2. Immune markers of 63 parameters**

| **Immune cell subsets** | **Marker** |
| --- | --- |
| T cell subsets | |
| T cells | CD3^+^ |
| Naïve CD4^+^ T cells | CD3^+^CD4^+^CD45RA^+^CCR7^+^ |
| Terminal differentiated CD4^+^ T cells | CD3^+^CD4^+^CD45RA^+^CCR7^-^ |
| Central memory CD4^+^ T cells | CD3^+^CD4^+^CD45RA^-^CCR7^+^ |
| Effective memory CD4^+^ T cells | CD3^+^CD4^+^CD45RA^-^CCR7^-^ |
| Exhausted of CD4^+^ T cells | CD3^+^CD4^+^CD28^-^ |
| Functional CD4^+^ T cells | CD3^+^CD4^+^CD28^+^ |
| Treg cells | CD3^+^CD4^+^CD25^+^CD127^-^ |
| Killer T cells | CD3^+^CD8^+^ |
| Naïve CD8^+^ T cells | CD3^+^CD8^+^CCR7^+^CD45RA^+^ |
| Terminal differentiated CD8^+^ T cells | CD3^+^CD8^+^CCR7^-^CD45RA^+^ |
| Exhausted CD8^+^ T cells | CD3^+^CD8^+^CD28^-^ |
| Terminally senescent CD8^+^ T cells | CD3^+^CD8^+^CD28^-^CD57^+^ |
| Inactive specific CD8^+^ T cells | CD3^+^CD8^+^CCR7^-^CD45RA^-^CD127^+^ |
| Inactive specific terminal differentiated CD8^+^ T cells | CD3^+^CD8^+^CCR7^-^CD45RA^+^CD127^+^ |
| Specific sustained expressed virus CD8^+^ T cells | CD3^+^CD8^+^CCR7^-^CD45RA^-^CD127^-^ |
| Specific sustained expressed virus terminal differentiated CD8^+^ T cells | CD3^+^CD8^+^CCR7^-^CD45RA^+^CD127^-^ |
| Total memory CD8^+^ T cells | CD3^+^CD8^+^HLADR^+^ |
| Homing memory CD8^+^ T cells | CD3^+^CD8^+^HLADR^＋^CD38^+^ |
| Central memory CD8^+^ T cells | CD3^+^CD8^+^CCR7^+^CD45RA^-^ |
| Effective memory CD8^+^ T cells | CD3^+^CD8^+^CCR7^-^CD45RA^-^ |
| Inhibitory CD8^+^ T cells | CD3^+^CD8^+^PD-1^+^ |
| Potential functional CD8^+^ T cells | CD3^+^CD8^+^CD28^+^ |
| Double positive T lymphocytes | CD3^+^CD4^+^CD8^+^ |
| Helper T cells | CD3^+^CD4^+^ |
| Peripheral T helper cells | CD3^+^CD4^+^CXCR5^-^PD-1^+^ |
| Th1 | CD3^+^CD4^+^CXCR5^-^CXCR3^+^CCR4^-^ |
| Th2 | CD3^+^CD4^+^CXCR5^-^CXCR3^-^CCR4^+^ |
| Th17 | CD3^+^CD4^+^CXCR5^-^CXCR3^-^CCR4^-^CCR6^+^ |
| Th1/Th2 | Ratio |
| Th17/Th2 | Ratio |
| (Th1^+^Th17)/Th2 | Ratio |
| Tfh | CD3^+^CD4^+^CXCR5^+^ |
| Activated follicular T helper cells | CD3^+^CD4^+^CXCR5^+^PD-1^+^ |
| Tfh1 | CD3^+^CD4^+^CXCR5^+^CXCR3^+^CCR4^-^ |
| Tfh2 | CD3^+^CD4^+^CXCR5^+^CXCR3^-^CCR4^+^ |
| Tfh17 | CD3^+^CD4^+^CXCR5^+^CXCR3^-^CCR4^-^CCR6^+^ |
| Tc1 | CD3^+^CD8^+^CXCR5^-^CXCR3^+^CCR4^-^ |
| Tc2 | CD3^+^CD8^+^CXCR5^-^CXCR3^-^CCR4^+^ |
| Tc17 | CD3^+^CD8^+^CXCR5^-^CXCR3^-^CCR4^-^CCR6^+^ |
| Th/Tc | Ratio |
| NK cell subsets | |
| TNK cells | CD3^+^CD56^+^ |
| NK cells | CD3^-^CD56^+^ |
| Immature NK cells | CD3^-^CD56^+/hi^ |
| Mature NK cells | CD3^-^CD56^+/lo^ |
| Early inhibition of NK cells | CD3^-^CD56^+^CD94^+^KIR^-^ |
| Late inhibition of NK cells | CD3^-^CD56^+^CD94^-^KIR^+^ |
| Activated NK cells | CD3^-^CD56^+^NKG2D^+^ |
| Conventional killer NK cells | CD3^-^CD56^+^NKP30^+^ |
| Specific virus killer NK cells | CD3^-^CD56^+^NKP46^+^ |
| Immature NK/mature NK cells | Ratio |
| γδT cells subsets | |
| γδT cells | CD3^+^gammadelta^+^ |
| Vδ1^+^ γδT cells | CD3^+^gammadelta^+^Vdelta2^-^ |
| Vδ2^+^ γδT cells | CD3^+^gammadelta^+^Vdelta2^+^ |
| Vδ2^+^NKG2D^+^ γδT cells | CD3^+^gd^+^Vdelta2^+^NKG2D^+^ |
| Vδ2^+^PD-1^+^ γδT cells | CD3^+^gd^+^Vdelta2^+^PD1^+^ |
| Vδ2^+^NKP30^+^ γδT cells | CD3^+^gd^+^Vdelta2^+^NKP30^+^ |
| Vδ2^+^NKP46^+^ γδT cells | CD3^+^gd^+^Vdelta2^+^NKP46^+^ |
| Vδ1^+^NKG2D^+^ γδT cells | CD3^+^gd^+^Vdelta1^+^NKG2D^+^ |
| Vδ1^+^PD-1^+^ γδT cells | CD3^+^gd^+^Vdelta1^+^PD1^+^ |
| Vδ1^+^NKP30^+^ γδT cells | CD3^+^gd^+^Vdelta1^+^NKP30^+^ |
| Vδ1^+^NKP46^+^ γδT cells | CD3^+^gd^+^Vdelta1^+^NKP46^+^ |
| Vδ1^+^ γδT / Vδ2^+^ γδT cells | Ratio |
