# Supplementary material for: Pregnancy Induces an Immunological Memory Characterized by Maternal Immune Alterations Through Specific Genes Methylation
Source: Front Immunol. 2021 Jun 7;12:686676. doi: 10.3389/fimmu.2021.686676 (PMC8215664; doi:10.3389/fimmu.2021.686676)
Supplement: Supplementary file 4 [file Table_3.docx]

**Table S3. Changed immune cell subsets in the NPW, PW and PE group**

| **Immune cell subsets** | **NPW**  **(n = 50)** | **PW**  **(n = 50)** | **PE**  **(n = 14)** |
| --- | --- | --- | --- |
| Helper T cells (%) | 59.02 ± 11.54 | 53.86 ± 9.97^*^ | 56.99 ± 7.53 |
| CD4^+^ T_CM_ cells (%) | 8.09 ± 3.95 | 8.71 ± 4.92 | 10.84 ± 4.58^*^ |
| Total memory CD8^+^ T cells (%) | 3.32 ± 2.13 | 3.42 ± 2.06 | 5.69 ± 4.00^**^ |
| Homing memory CD8^+^ T cells (%) | 58.12 ± 17.05 | 50.91 ± 14.71^*^ | 62.46 ± 13.45 |
| Th2（%） | 18.06 ± 6.17 | 15.08 ± 4.95^**^ | 15.32 ± 3.96 |
| Tc2（%） | 5.94 ± 3.59 | 4.51 ± 2.67^*^ | 5.17 ± 2.63 |
| Tc17（%） | 8.80 ± 6.21 | 7.49 ± 7.60 | 4.63 ± 3.99^*^ |
| Peripheral helper T cells (%) | 60.97 ± 5.88 | 57.13 ± 3.76^***^ | 62.08 ± 4.47 |
| Immature NK cells (%) | 77.18 ± 22.00 | 75.68 ± 17.13 | 45.52 ± 31.38^***^ |
| Mature NK cells (%) | 22.30 ± 22.04 | 23.76 ± 16.80 | 54.63 ± 31.56^***^ |
| Immature/mature NK cells | 8.90 ± 7.80 | 5.80 ± 5.16^*^ | 2.10 ± 2.88^**^ |
| Vδ1^+^γδT cells (%) | 43.23 ± 21.18 | 43.09 ± 19.92 | 67.84 ± 26.59^***^ |
| Vδ2^+^γδT cells | 56.69 ± 21.20 | 56.81 ± 19.93 | 32.05 ± 26.59^***^ |
| Vδ1^+^/ Vδ2^+^ γδT cells | 1.18 ± 1.31 | 1.13 ± 1.33 | 13.38 ± 33.02^**^ |
| Vδ2^+^NKG2D^+^ γδT cells (%) | 96.93 ± 4.80 | 93.93 ± 9.09^*^ | 99.60 ± 0.67^*^ |
| Vδ2^+^PD-1^+^ γδT cells (%) | 9.03 ± 7.77 | 7.82 ± 6.99 | 14.72 ± 7.82^**^ |
| Vδ2^+^NKP30^+^ γδT cells (%) | 1.15 ± 1.65 | 2.31 ± 2.56^**^ | 4.64 ± 3.76^***^ |

Data are analyzed by Mann-Whitney U test, and presented as Mean ± SEM. Compared with NPW group: **P* < 0.05; ***P* < 0.01; ***P<0.001.
