# Supplementary material for: Pregnancy Induces an Immunological Memory Characterized by Maternal Immune Alterations Through Specific Genes Methylation
Source: Front Immunol. 2021 Jun 7;12:686676. doi: 10.3389/fimmu.2021.686676 (PMC8215664; doi:10.3389/fimmu.2021.686676)
Supplement: Supplementary file 5 [file Table_4.docx]

**Table S4. All DMGs in the PW and the PE group**

| **Groups** | **Hyper-methylated genes** | **Hypo-methylated genes** |
| --- | --- | --- |
| PE *vs.* NPW | FGF1 | RUNX1 |
|  | CASP7 | PITX2 |
|  | HIST1H4L | CLDN15 |
|  | UNC13A | NR2E1 |
|  | TRPV2 | SPP1 |
|  | OR6C70 | GNAQ |
|  | SSTR2 | RPL29 |
|  | OR10X1 | UBE2F |
|  | IST1 | RPL11 |
|  | TUBA1C | TNFSF13B |
|  | PANK3 | GSPT1 |
|  | OR1G1 | CA5A |
|  | AGTR1 | SLC8A1 |
|  | SNX2 | GNAS |
|  | ADCY10 | PISD |
|  | SIPA1L2 | NMD3 |
|  | GAL3ST1 | CABP1 |
|  | GAPDHS | PFKFB3 |
|  | SEC31B | KCNMB1 |
|  | CNTN1 | YWHAZ |
|  | DNAH5 | HTR3B |
|  | MOCS3 | DDX23 |
|  | PPARD | NUP160 |
|  | KCNE1 | MSH3 |
|  | GNG7 | DUSP6 |
|  | SCLY | WWP2 |
|  | LPP | CD7 |
|  | VPS26A | PPCDC |
|  | BPGM | PMAIP1 |
|  | PSMB5 | CCND3 |
|  | MRC1 | RPS26 |
|  | TRPM2 | FLT3 |
|  | GMDS | ATF1 |
|  | SF3A2 | SEMA5B |
|  | CYP3A43 | CDC23 |
|  | FXN | LNPEP |
|  | GRHPR | AHCY |
|  | AP3M2 | TK1 |
|  | TAB2 | TOB2 |
|  | GSR | CSF1R |
|  | CD6 | BTD |
|  | ABLIM1 | NFKBIZ |
|  | B3GAT2 | PLA2G12B |
|  | APC | UNC13C |
|  | GRAP2 | RGPD2 |
|  | MRPL28 | NCK1 |
|  | WEE1 | PRKG2 |
|  | ALG2 | AUH |
|  | AMBRA1 | ANGPT2 |
|  | RAB7A | APP |
|  | B3GALT1 |  |
|  | CHST4 |  |
|  | TPM4 |  |
|  | ECHS1 |  |
|  | GSTM2 |  |
|  | CADM1 |  |
|  |  |  |
| PW *vs.*NPW | SLC7A7 | PSMC5 |
|  | MET | URM1 |
|  | TFRC | SLC51A |
|  | HIST1H4K | GATM |
|  | PROCR | GNAS |
|  | BRK1 | ISYNA1 |
|  | FADS6 | FZD3 |
|  | MAN2C1 | IL21R |
|  | E2F5 | RAMP3 |
|  | NAGK | RAP1A |
|  | AMBRA1 | GAPDHS |
|  | USE1 | RPS16 |
|  | HYI | HMGCL |
|  | PAPSS1 | FYB1 |
|  | AKAP8L | SLC8A1 |
|  | CUL2 | PDE4A |
|  | APH1B | ATF6 |
|  | SFRP5 | SRSF1 |
|  | NUMB | CD247 |
|  | TFAP4 | UBE2L6 |
|  | PRICKLE2 | NUP160 |
|  | CAPZA2 | DEGS1 |
|  | PRPH | CLDN25 |
|  | MAN2A2 | HCAR3 |
|  | SLU7 | UGT1A5 |
|  | BCAT2 | GRAP2 |
|  | CYB5R3 | PIK3AP1 |
|  | UQCRFS1 | GNA15 |
|  | SETDB1 | GABRP |
|  | GEMIN4 | DNAJB1 |
|  | SF3B1 | CD86 |
|  | LYPLA1 | NANS |
|  | RGPD2 | NPBWR1 |
|  | AP1M1 | PMS2CL |
|  | IQSEC1 | NUP85 |
|  | SPTLC1 | ETS1 |
|  | GSTK1 | CNGB3 |
|  | ACKR1 | PPP2R2C |
|  |  | LBP |
|  |  | ERCC6 |
|  |  | PLA2R1 |
|  |  | GADD45B |
|  |  | TRIM71 |
|  |  | CLDN18 |
|  |  | LSM6 |
|  |  | CLDN10 |
|  |  | GANC |
|  |  | ACER2 |
|  |  | RPL13 |
|  |  | KCNJ8 |
|  |  | RANBP2 |
|  |  | BTRC |
|  |  | OR1L1 |
|  |  | ANK2 |
|  |  | SAT2 |
|  |  | MDN1 |
|  |  | SRSF3 |
|  |  | PGA3 |
|  |  | PILRA |
|  |  | SNX3 |
|  |  | DAGLA |
|  |  | SLCO1B3 |
|  |  | ESR1 |
|  |  | ACSF3 |
